# Development of Multifunctional Slag and Bauxite Residue-Based Geopolymers with Heavyweight Aggregate Enhancement

**DOI:** 10.3390/ma18174087

**Published:** 2025-09-01

**Authors:** Andrie Harmaji, Reza Jafari, Guy Simard

**Affiliations:** Department of Applied Sciences, University of Québec in Chicoutimi (UQAC), 555, boul. de l’université, Saguenay, QC G7H 2B1, Canada

**Keywords:** geopolymer, Fe-rich spinel aggregate, electrical conductivity, photothermal, acid resistance

## Abstract

The growing demand for sustainable and multifunctional construction materials, particularly those capable of addressing durability and energy challenges, has motivated the development of conductive and photothermally active geopolymers. This study investigated the use of an Fe-rich spinel aggregate (FSA) as a high-density filler in geopolymers composed of ground granulated blast furnace slag and bauxite residue, with a fixed addition of 1 wt% graphite (binder-based) to enhance electrical conductivity. The effects of different FSA replacement percentages (0–100%) on compressive strength, electrical conductivity, photothermal efficiency, and chemical resistance were evaluated. An increase in the FSA content translated to an increase in the final compressive strength, with 100% FSA replacement achieving the highest value of 45.5 ± 2.5 MPa at 28 days. As the FSA content increased, the electrical resistivity decreased to as low as 42 Ω·m at 100% replacement. Under simulated solar flux conditions (1 kW/m^2^), photothermal analysis revealed that the 100% FSA mixtures exhibited the highest surface temperature increase of 9.8 °C after 300 s, indicating their superior thermal responsiveness. Furthermore, acid immersion in 10% HCl for 28 days showed mass gain in all geopolymers, with the highest gain observed at 50% FSA (+11.51%). Similarly, the strength increased after acid exposure up to a 75% FSA content. These findings highlight the multifunctional potential of FSA-enhanced geopolymers for high-mechanical-performance, electrically conductive, photothermally active, and chemically durable materials as multifunctional construction materials.

## 1. Introduction

The advancement of technology, coupled with the increasing global energy demands, has driven a transition away from fossil fuel-based power sources toward cleaner, low-carbon alternatives, such as nuclear energy. Concurrently, the global use of radiation-emitting devices has increased, raising significant health concerns [1]. Heavyweight concrete is commonly utilized for shielding applications owing to its economical aspect and good mechanical properties [2]. This special type of concrete is made using heavyweight aggregates, which are important in improving radiation-blocking performance owing to their good attenuation capacity against photons and neutrons [3]. The common aggregates used to develop heavyweight concrete are magnetite, hematite, ilmenite, and barite [4]. One application of heavyweight concrete is hazardous waste containment [5]. For this purpose, high acid resistance is essential, as acid leaching can degrade the protective barrier, potentially resulting in contaminant leakage or structural failure. However, one main raw material for creating heavyweight concrete is Portland cement, which has poor resistance to an aggressive acidic environment [6]. Moreover, Portland cement production is a significant source of global carbon emissions, as it is responsible for approximately 7–8% of the total global carbon emissions [7].

Owing to these factors, newer and more eco-friendly construction materials are in high demand. One more promising alternative is geopolymers, which utilize the byproducts of various industries, such as ground granulated blast furnace slag (GGBFS) [8] and bauxite residue [9], as the main raw materials. GGBFS is an industrial byproduct that is commonly reused in cement and concrete production, whereas bauxite residue is still largely regarded as waste and disposed of by various industries [10]. Recent studies have highlighted the anticipated benefits of geopolymers over ordinary Portland cement (OPC) concrete [11]. For example, geopolymers have shown the potential to replace OPC concrete by reducing up to 65% carbon emissions with the same strength grades [12]. Expanding on these benefits, Zhang et al. [13] found that the acid resistance of geopolymers can be improved by incorporating siliceous materials. Furthermore, when exposed to acidic environments, geopolymers have demonstrated notable corrosion resistance compared with OPC-based concrete [14]. However, it has been reported that lightweight geopolymer are not durable in sulfate environments, such as oil-well cementing [15].

Fe-rich spinel aggregate (FSA) is a promising conductive aggregate owing to its multifunctional properties and prevalence in high-specific-gravity fillers for heavyweight concrete applications. This material is composed of naturally occurring spinel-type minerals, which are engineered for enhanced performance, including shielding capacity, conductivity, and thermal performance. FSA is characterized by a dark color and relatively low resistivity due to its metallic iron content [16], in addition to its heat transfer capability. Furthermore, it can be tailored to the needs of specific applications, benefiting structural and functional applications, such as energy storage [17]. Originally developed as a photothermal material, its high capability of light absorption enables a conversion to thermal energy, which broadens its range of potential functionalities [18]. Given the already broad applicability of heavyweight concrete in radiation shielding of research facilities, protective barriers, counterweights for cranes, ballast for docks, and underground structures, the development of an FSA-enhanced alkali-activated material represents a sustainable pathway for advanced functional infrastructure.

However, FSA has not yet been investigated as a partial or complete replacement for fine aggregates in alkali-activated systems. Therefore, this study focused on developing a heavyweight composite based on alkali-activated slag and bauxite residue, incorporating FSA to enhance its functional properties. The properties of the geopolymer were evaluated in terms of compressive strength, electrical conductivity, photothermal response, chemical stability, phase composition using X-ray diffraction (XRD), and microstructural characteristics using scanning electron microscopy (SEM).

## 2. Materials and Methods

### 2.1. Materials

#### 2.1.1. Aluminosilicate Sources

The bauxite residue used in this study was provided by the Canadian alumina refinery Rio Tinto in Jonquière, QC, Canada. The material was utilized without any prior processing, such as oven drying or neutralization. Therefore, the appearance resembles clay with a particle size of 10–200 µm and coagulated granularity. GGBFS was produced by Lafarge cement in St. Constant, QC, Canada. The oxide compositions of the bauxite residue and GGBFS were determined using X-ray fluorescence (XRF) spectrometry by Bureau Veritas Laboratories, Saint-Laurent, QC, Canada in Table 1.

#### 2.1.2. Alkali Activator

Sodium hydroxide (NaOH) crystals (77.5% Na_2_O and 22.5% H_2_O) were obtained from Laboratoire MAT (Québec City, QC, Canada). It was then mixed with tap water to produce a solution of 16 mol/L. The sodium silicate solution (8.9% Na_2_O, 28.7% SiO_2_, and 62.4% H_2_O) was obtained from Univar Solutions (North York, ON, Canada). During the experiment, a sodium hydroxide solution was added to adjust the SiO_2_-to-Na_2_O ratios of the sodium silicate solution. The two solutions were combined, thoroughly stirred, and allowed to cool down for 6 h before mixing with aluminosilicate, in accordance with the method used in a previous study [19]. This method was adopted because the mixing of NaOH and Na_2_SiO_3_ solutions can release heat, which can induce problems such as efflorescence in the latter stage and reduced compressive strength. The exothermic reaction when mixing NaOH and Na_2_SiO_3_ may release heat that can cause flash set [20]. Some alkali may not fully reacted with aluminosilicate and can react with CO_2_ instead to form a sodium carbonate phase such as Na_2_CO_3_ or NaHCO_3_ [21], some of the known efflorescence compounds. Therefore, controlling the mixing conditions is essential to ensure uniform geopolymerization and maintain material performance.

#### 2.1.3. Aggregates

Natural sand as a fine aggregate was obtained from a quarry in Saint-Henri-de-Taillon, Canada, to produce Portland cement-based concrete and alkali-activated material (AAM) mortar. The absorption coefficient and fineness modulus of the sand were 0.28% and 2.94, respectively. The materials complied with the Canadian Standards Association (CSA) A23.1-19 [22], “Concrete Materials and Methods of Concrete Construction”, requirements. The fine FSA used to produce heavyweight AAM was provided by Magnor Exploration (Saguenay, QC, Canada). It had an absorption coefficient of 1.68% and a specific gravity of 5.0 g/cm^3^.

#### 2.1.4. Graphite

In this study, 1 wt% (binder-based) graphite was included to promote the electrical conductivity of the geopolymer while enhancing workability and mechanical performance. The graphite (99% purity) was obtained from Fisher Scientific (Ottawa, ON, Canada). Graphite is an inexpensive and commonly used conductive additive that can facilitate the movement of electrons through the matrix to lower electrical resistivity and optimize performance for applications, including indoor radiant heating [23] and snow-melting concrete [24]. Research has demonstrated the conductivity benefits of 1–4 wt% graphite to improve the conductivity of cementitious materials while not excessively compromising their fresh or hardened properties [25]. Excessive dosages (>1 wt%) of graphite can increase the water demand and induce agglomeration, thus compromising the distribution of the conductive agent and mechanical performance of the slurry [26]. Therefore, 1 wt% graphite was determined to be the most practical amount added to optimize electrical performance and workability while maintaining the structural integrity of the geopolymer.

### 2.2. Methods

#### 2.2.1. Mix Design Proportions

Six mixture proportions were designed to compare standard concrete and the geopolymer. An air-entrained concrete (AEC) that conformed to the CSA A23.1-19 [22] requirements was prepared as the reference sample (Table 2). The AEC consisted of cement, fine and coarse aggregates, Sika Air 60 air-entraining agent, and Sika Viscocrete (high-range water reducing and superplasticizing) admixture. Air entrainment was selected as it is common in Canada because the infrastructure is exposed to some of the worst freeze–thaw conditions, and the entrained air voids provide the necessary resistance to frost damage due to freezing water [27].

The mix design of bauxite residue and GGBFS-based geopolymers, including the curing method, was designed on the basis of previous research [28]. The fine FSA was used as a partial substitute for the fine aggregate in the amounts of 0%, 25%, 50%, 75%, and 100%. The complete mixture is presented in Table 3. A planetary mixer was used to mix the binder, aggregate, graphite, and alkali activator to form a slurry. The slurry was then cast into the mold, followed by ambient curing at room temperature for 7 and 28 days. Each specimen was covered with a hydrophobic film to prevent moisture loss throughout the curing process.

#### 2.2.2. Slump Flow and Bulk Density

**Figure 1:**
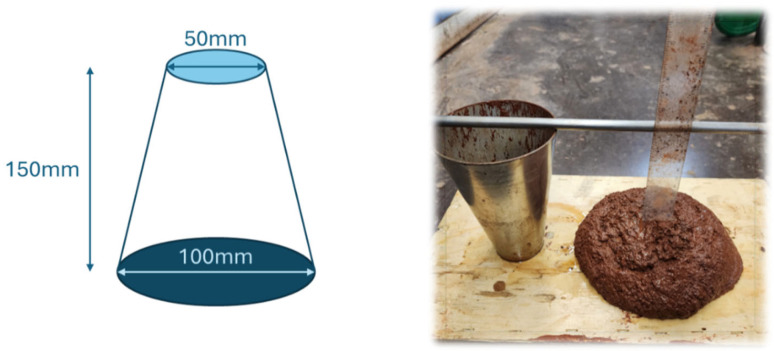
Mini-MBE cone test for measuring the slump of mortar.

The slump of the mortar was measured immediately after mixing using a mini MBE cone, which evaluates the consistency on the basis of the ratio of the solid surface area to the fluid content [29], as shown in Figure 1. A slump cone made of steel plates was filled with fresh geopolymer mortar. The mortar was then poured out freely as the slump cone was raised vertically. The slump is defined as the height differential between the mold and the fresh mortar, and the bulk density was calculated using the weight measurement of the hardened geopolymer.

#### 2.2.3. Compressive Strength Test

A 50 mm × 50 mm × 50 mm cubical sample was made in accordance with the CSA A23.2-9C [22], “Method of Test for Compressive Strength of Hydraulic Cement Mortars”, requirements. After 7 and 28 days of ambient curing, compressive strength tests were performed on the sample. The compressive tests were carried out using a CONTROLS PILOT PRO-EN Automatic Compression Testers machine in Saguenay, QC, Canada with a loading rate of 0.25 ± 0.05 MPa/s that was maintained until the failure of the specimen, with the failure point being the maximum load that the test specimen could withstand before collapsing or fracturing. The mean values were used as representative strengths.

#### 2.2.4. Electrical Property Tests

**Figure 2:**
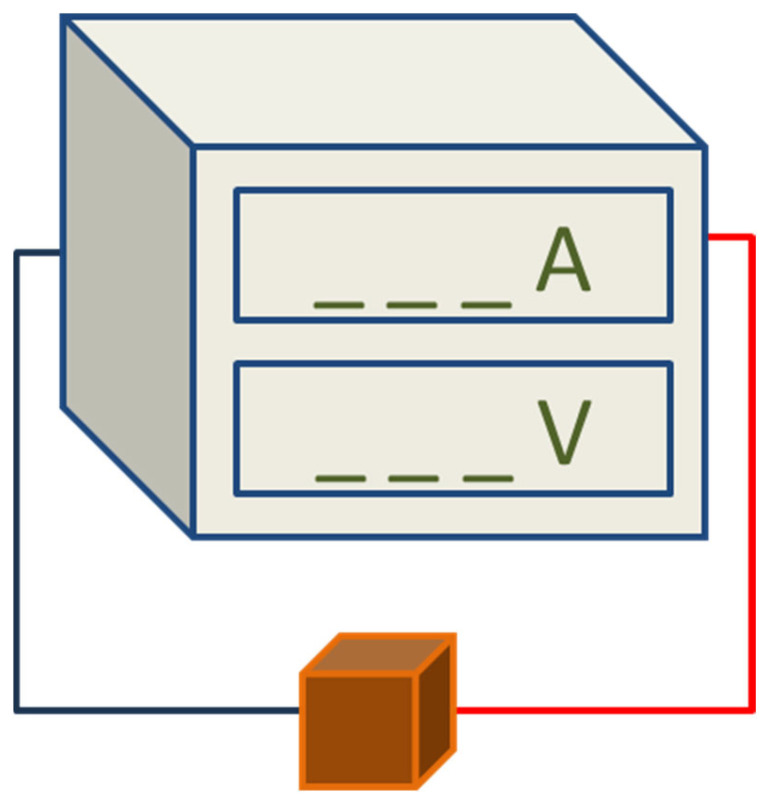
Schematic illustration of the experimental setup for determining the electrical conductivity of the geopolymer.

To measure the electrical properties, the heavyweight geopolymer specimen dimensions were maintained at 50 mm × 50 mm × 50 mm, consistent with the same dimensions used for the compression tests. To ensure reliable contact with the electrodes, the surface was polished with 1000-grit silicon carbide and coated with carbon black powder (≥99% purity; Fisher Scientific, Ottawa, ON, Canada). The two-probe method was used to measure the resistivity of the specimens, as it is a relatively easy way to measure the electrical properties of materials and has been widely used [30]. The specimen was enclosed in a Faraday cage to reduce the effects of external electromagnetic interference. DC conductivity was determined using an HIOKI P5335 Power Meter programmable voltage source (Figure 2).

The samples were subjected to an applied voltage of 0 to 150 V in 30 V steps. The current (*I*) and voltage (*V*) were then recorded. The heat flux (*Q*, in W/m^2^) was calculated using Ohm’s Law, as in Equation (1):

(1) $$Q=\;\frac{V\;\times I}{A}$$

#### 2.2.5. Photothermal Performance

**Figure 3:**
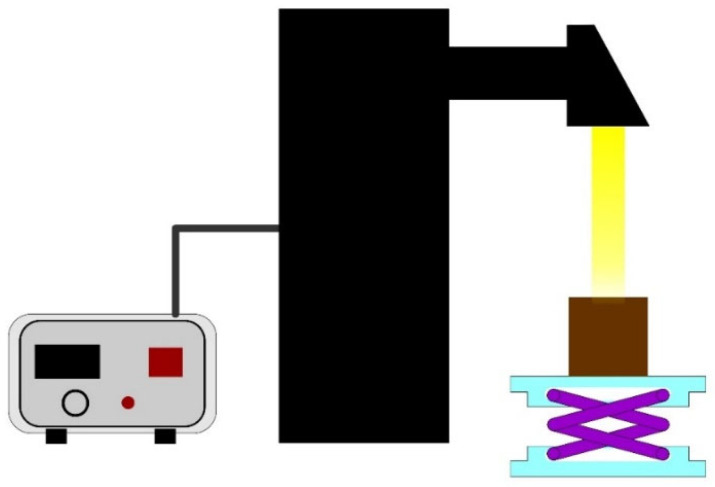
Schematic illustration of the experimental setup for determining the photothermal conversion performance.

To investigate the photothermal conversion performance of the heavyweight geopolymer, the 50 mm × 50 mm × 50 mm cubical samples were exposed to a sunlight simulator (Figure 3). The temperature change was measured at the following points after exposure to a photothermal flux under one solar light intensity (1 KW/m^2^): 0, 60, 120, 180, 240, and 300 s. This value was adopted in line with previous studies [31] on the photothermal performance of geopolymers.

#### 2.2.6. Chemical Resistance

To determine the degrading behavior of the samples in a solution of acids to simulate a harsh real-life environment, geopolymer samples with similar dimensions for compressive strength (50 mm mortar cubes) were utilized for each mix. The specimens were cast, coated with plastic, and stored at room temperature for a day. Afterward, the specimens were left at ambient temperature for the remaining 24 days before testing for acid resistance. The specimens were initially immersed in 10% hydrochloric acid (HCl) solutions (2.87 mol/L) with a pH of 1  ±  0.1 to simulate intense acid environments, such as the food industry, mining, sewer pipes, and nuclear power plants [32]. This concentration was selected based on previous geopolymer studies where 10% HCl has been employed to evaluate durability [33]. The resistance of the specimens to acid attack was measured in terms of mass and compressive strength loss after a soaking time of 28 days. The mass of the specimens before immersion in the acid solution was measured as the initial mass (*m_i_*). After the immersion at 28 days of age, the samples were rinsed with tap water and wiped down using a damp towel. The mass of the samples was recorded as the residual mass (*m_r_*). The mass loss percentage due to acid immersion was calculated using Equation (2):

(2) $$Mass\;loss=\;\frac{m_{r}-m_{i}}{m_{i}}\;\times 100$$

#### 2.2.7. XRD Characterization

**Figure 4:**
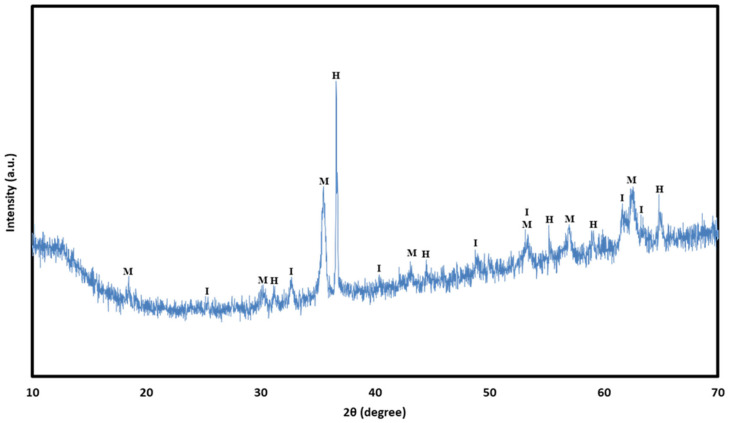
XRD patterns of the Fe-rich spinel aggregate (H, hercynite; I, ilmenite; M, magnetite).

The chemical behaviors of the AEC and geopolymer were examined using XRD (Rigaku Smartlab, Tokyo, Japan). The analysis was performed with scanning angle from 5° to 80° (2θ) at a rate of 2.5°/min over a total acquisition time of 30 min. The characterization was conducted to 28-day samples of raw materials and hardened samples (AEC and geopolymers) after 28 days. To fit the sample holder, the materials were powdered with a mortar and pestle. Figure 4 reveals the diffractogram of FSA, which is predominantly composed of hercynite (FeAl_2_O_4_) with lower amounts of magnetite (Fe_3_O_4_) and ilmenite (FeTiO_3_). The dominance of hercynite with its spinel (AB_2_O_4_) structure, together with magnetite, justifies referring to the material as an FSA.

**Figure 5:**
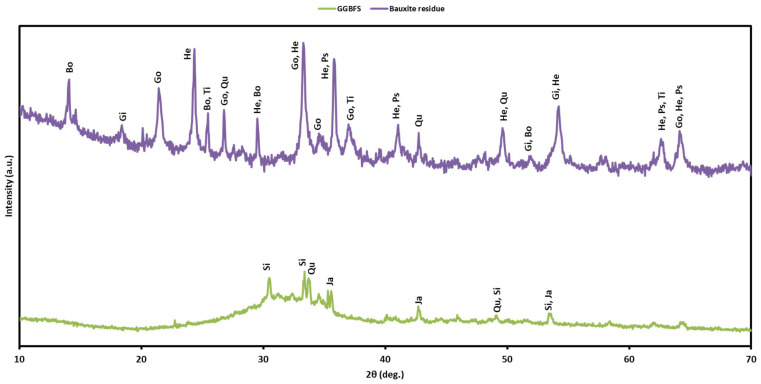
XRD patterns of the bauxite residue and GGBFS (Si—calcium silicate, Q—quartz, Ja—jadeite, He—hematite, Gi—gibbsite, Go—goethite, Bo—boehmite, Ti—anatase, Ps—pseudorutile).

In Figure 5, the bauxite residue indicates the formation of boehmite (Al_2_O_3_H_2_O), gibbsite (Al(OH)_3_), goethite (FeO(OH)), anatase (TiO_2_), pseudorutile (Fe_2_Ti_3_O_9_), quartz (SiO_2_), and hematite (Fe_2_O_3_) as the main crystalline phases. Subsequently, the GGBFS is mainly composed of quartz, calcium silicate (Ca_2_SiO_4_), and jadeite (NaAlSi_2_O_6_). Based on the XRD diffractogram, the GGBFS exhibits a predominantly amorphous phase, indicating its high reactivity and suitability as a raw material for geopolymer synthesis.

#### 2.2.8. SEM Characterization

The morphologies of the AEC and geopolymer were investigated using a scanning electron microscope (SEM; JEOL JSM-7800 F, Tokyo, Japan). SEM observations were performed in high-vacuum mode at a 12 mm working distance, and images were captured at 1000× magnifications to examine the change before and after chemical exposure. For the microstructural analysis, fragments obtained from the compressive strength test were collected, and their cross-sections were prepared for SEM examination.

## 3. Results and Discussion

### 3.1. Slump Flow and Bulk Density of Fresh Mortars

**Figure 6:**
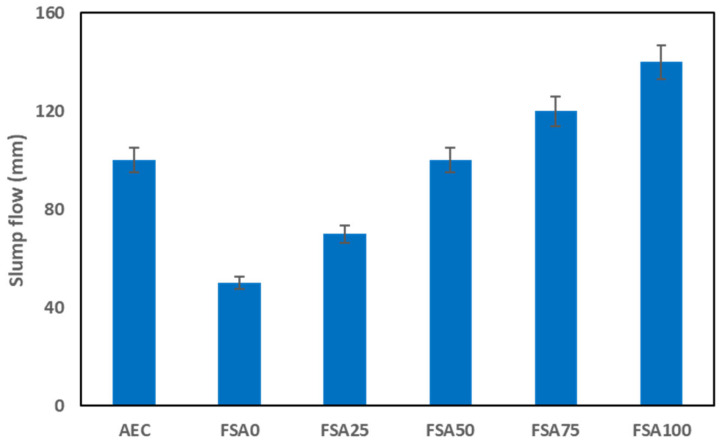
Influence of the replacement of sand with FSA on slump flow.

Slump flow is a good indicator of the workability and other features of fresh and hardened mortars [34]. The AEC and geopolymer flowability data are displayed in Figure 6. The results show an increasing flow spread with increasing replacement of FSA. The mixture fluidity of FSA0 was measured at 50 mm. As the aggregate replacement increased to 25%, 50%, 75%, and 100%, the slump flow values were recorded at 70, 100, 120, and 140 mm, respectively. The flow spread of 50% FSA replacement was 100 mm, which is well within the range of the properties that resemble AEC mixes.

The increasing flow spread with increased magnetite replacement can be explained by the higher density and lower water absorption of magnetite than those of sand [35,36]. Less water adsorption and a greater density mean less overall surface area and reduced internal friction between the solid particles in the mixture, which translates into enhanced flowability. In addition, the increased density of magnetite allowed the mix to spread out more freely than a sand-only mix under the force of gravity, thereby improving fresh-state performance. This result is in contrast to other research showing that magnetite aggregates decrease the slump of a fly ash, GGBFS, and silica fume-based geopolymer [37].

**Figure 7:**
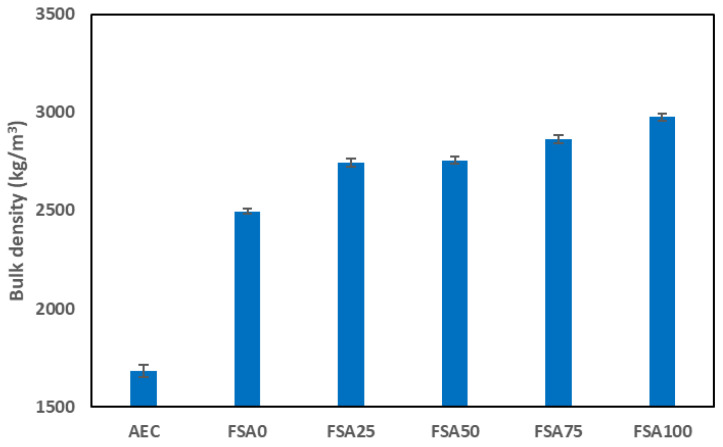
Effect of the replacement of sand with FSA on bulk density.

The bulk density of the geopolymers was measured at different magnetite replacement levels to evaluate the impact of high-density aggregate substitution, as displayed in Figure 7. The results show a significant increase in density as the proportion of FSA increased.

The mix without FSA showed a density of 2216 ± 4.5 kg/m^3^, whereas replacing 25%, 50%, 75%, and 100% of sand with the heavyweight aggregate resulted in densities of 2543 ± 4.5, 2798 ± 5.5, 2811 ± 2.5, and 3188 ± 1.9 kg/m^3^, respectively. These results are in line with other research, which revealed that the use of magnetite as a coarse aggregate replacement in a fly ash and GGBFS geopolymer increased its bulk density [37]. In contrast, a typical AEC has a considerably lower mass per unit volume of 1683 ± 3.3 kg/m^3^, highlighting the substantial mass increase due to the FSA content. The increase in such physical properties can be attributed to the high specific gravity of the FSA, which contains dense mineral phases of magnetite, ilmenite, and hercynite compared with quartz-based sand (approximately 2.65 g/cm^3^) [38]. As the content increases, the overall particle packing efficiency improves, reducing internal voids and increasing mass per unit volume. The density increase is more pronounced from 0% to 50% FSA but stabilizes between 75% and 100% replacement, suggesting a saturation effect on the particle matrix. This could be due to the limitations in further compacting the matrix, as excess FSA may not significantly alter the internal structure of the mix beyond a certain threshold. The high weight concentration of the FSA-enhanced geopolymer makes it suitable for applications that require enhanced thermal mass, radiation shielding, and improved mechanical stability, while its workability trends must be carefully managed to maintain appropriate construction performance [39]. In addition, the increased density may influence transportation, handling, and structural load considerations, necessitating careful planning in practical applications.

### 3.2. Compressive Strength

**Figure 8:**
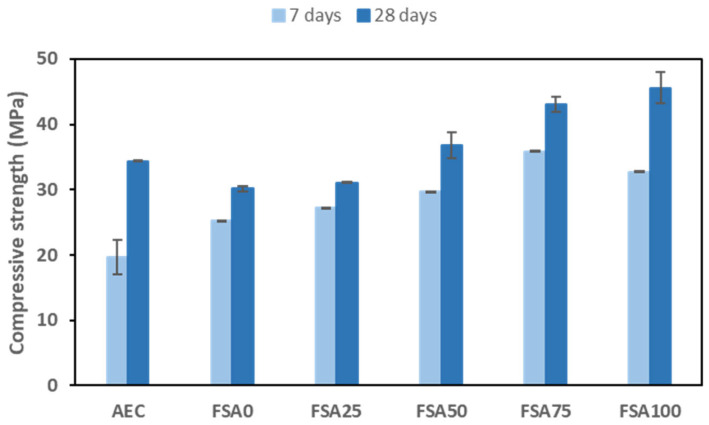
Impact of FSA as a partial replacement for the fine aggregate on the compressive strength of the geopolymer.

The 7- and 28-day compressive strengths of all mixtures in Table 2 and Table 3 are presented in Figure 8. In Portland cement-based mixes, the reference sample has 7- and 28-day strengths of 19.6 ± 2.6 and 34.4 ± 0.1 MPa, respectively. The hydration of the cement in the concrete mix causes the formation of calcium–silicate–hydrate (C-S-H) gel, which accounts for the increase in mechanical properties with age [40].

The mechanical performance of the geopolymer samples improved progressively with the higher FSA content used as a fine aggregate substitute in ambient curing. This method was selected to simulate field conditions and evaluate the practicality of FSA geopolymers without energy-intensive curing. The mix with a 0% FSA content had a compressive strength of 25.2 ± 2.6 MPa at 7 days and increased to 30.1 ± 0.4 MPa at 28 days. In the presence of 25% FSA, the mechanical properties increased slightly to 27.2 ± 0.1 MPa at 7 days and 31.09 ± 0.1 MPa at 28 days, indicating a negligible increase in early-age strength attributed to the increased density and compactness of the mix. Substituting the fine aggregate with 50% FSA had a much better result of a compressive strength of 29.6 ± 0.1 MPa at 7 days and 36.8 ± 1.9 MPa at 28 days, which can be explained by the densification effect of the aggregate replacement particles improving the packing and developing a more refined pore structure, with lower voids leading to higher mechanical properties [41]. Moreover, the very high Fe_2_O_3_ content of the FSA may also lead to secondary reaction products that strengthen the interfacial transition zone (ITZ), thus decreasing micro-cracking and increasing the bond between the aggregate and geopolymer gel.

A notable improvement in strength was attained at 75% replacement with FSA, with values reaching 35.1 ± 0.1 MPa at 7 days and 43.1 ± 1.1 MPa at 28 days. The increase in mechanical performance was the largest among all the mixtures in this study. This enhancement may be attributed to the higher specific gravity of the FSA, allowing for more interlocking between aggregates and the binder, thereby increasing load transfer mechanisms [37]. In addition, replacing the aggregate can introduce pozzolanic and thermal effects that may enhance long-term strength by modifying the reaction kinetics of the alkali activation process [42]. At full sand replacement (100%), the mix exhibited a 7-day strength of 32.8 ± 0.2 MPa, which was slightly below the 75% FSA mix, but it achieved the highest 28-day strength of 45.6 ± 2.5 MPa. Although early-age development is somewhat impaired, long-term curing improves the alkali activation process and the development of mechanical properties [43]. However, adding too much FSA could modify porosity and alter binder–aggregate interactions in ways that affect the microstructure of the matrix and the rate of strength gain [44]. While slower strength development and carbonation are possible in ambient curing, compressive strengths above 40 MPa were still achieved at 28 days. In previous studies [45], the incorporation of magnetite as additives into metakaolin-based geopolymers was found to reduce compressive strength as its content increased, which was attributed to a decrease in the dissolution reaction order, indicating the participation of alternative reaction mechanisms distinct from the typical MK dissolution kinetics.

Along with strength, the stiffness of the material, usually described by Young’s modulus, is also an important parameter for having a deeper understanding of the behavior and characteristics of a geopolymer [46]. Although Young’s modulus experimental data were not collected in this study, it is reasonable to assume that the variability in stiffness would follow a similar variation to that of compressive strength since matrix densification and interaction with the aggregate affect both properties of the geopolymer.

### 3.3. Electrical Performance

**Figure 9:**
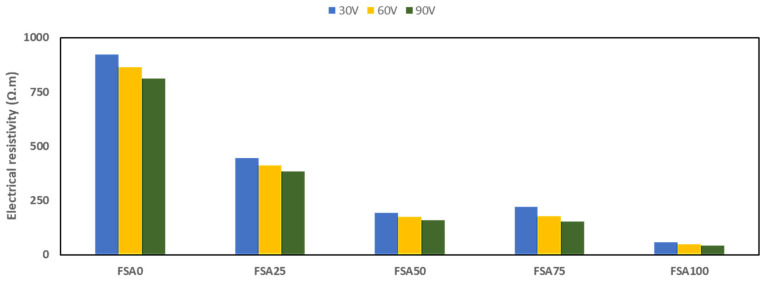
Influence of FSA as a partial replacement for fine aggregate on the electrical resistivity of the geopolymer.

The electrical resistivity of the geopolymer varied substantially through the partial replacement of sand with FSA (Figure 9). The observed conductivity results represent a synergistic effect of FSA and graphite.

The AEC mix is not included in the figure because its resistivity was too high to measure accurately within the test range, exceeding 36,000 Ω·m at 30 V. Among the geopolymer samples, the FSA0 mix had the lowest conductivity, which is usually exhibited by conventional geopolymers [47,48]. As the FSA content and applied voltage increased, a significant decrease in resistivity was observed. This change is most likely attributed to the inclusion of FSA, which provides more conductive pathways than insulating sand [49]. The greatest decrease in electrical resistance occurred in FSA100, which ranged from 42 to 57 Ω m. This trend indicates that FSA can aid in charge flow through the matrix of the concrete, possibly by improving particle-to-particle connectivity and reducing impedance [50]. A previous study supports these findings, where the semi-conductivity associated with magnetite-based aggregates translated to improved conductivity of cementitious composites via more interconnected conductive networks [51]. Percolation thresholds have been established for electrically conductive fillers [52]. In FSA-based systems, this is likely governed by the combined effects of electron hopping mechanisms and percolation theory, which also explains the nonlinear increase at higher replacement [53].

**Figure 10:**
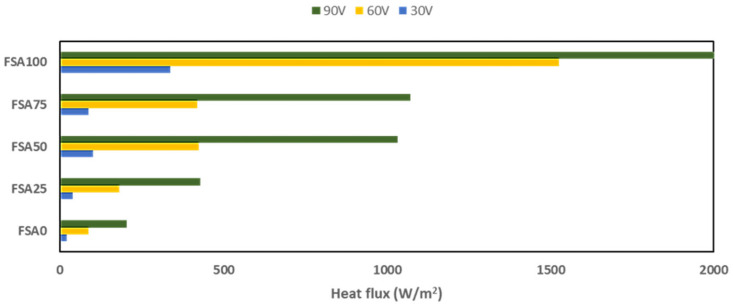
Consumed heat flux at different applied voltages for different specimens.

An Fe-rich aggregate-based cementitious material has prospective uses in conductive infrastructure applications, such as snow-melting pavements [54] and self-heating concrete [55]. It has been estimated that a surface heat flux between 300 and 550 W/m^2^ is needed to effectively melt snow and ice in cold climates [56,57]. The measured current, voltage, and surface area of the geopolymer specimens were used as inputs for heat flux calculations (Figure 10).

The calculation in Figure 10 indicated that the FSA0 mix did not achieve sufficient conductivity even at 90 V to meet the threshold required for snow-melting applications. This voltage requirement decreased when FSA was used to substitute the sand, with a power value higher than 300 W/m^2^ achieved at 90 V (FSA25), 60 V (FSA50 and FSA75), and 30 V (FSA100). A power density of 337 W/m^2^ was achieved by the latter using just 30 V. The results highlight the benefits of using FSA for the mix, as an increase in the aggregate replacement content tends to lower the voltage requirement for the geopolymer to achieve the power needed for snow-melting applications.

Although these results show encouraging heating performance at the laboratory scale, careful consideration of long-term durability through repeated freeze–thaw and heating–cooling cycles will be needed for real-world deployment, as thermal cycling may develop microcracking and change conductivity pathways [58]. It will equally be necessary to optimize the trade-off between energy input and heat output for energy efficiency, since rising applied voltages increase the heat of concrete [59]. Therefore, field-scale testing with life-cycle energy and cost assessments will be needed to determine the practicality of these applications in real-world contexts. Furthermore, while the two-probe DC method may introduce contact resistance, it was chosen for its simplicity and relevance to bulk electrical performance. Future work should employ four-probe or impedance spectroscopy for more precise characterization.

### 3.4. Photothermal Conversion Performance

**Figure 11:**
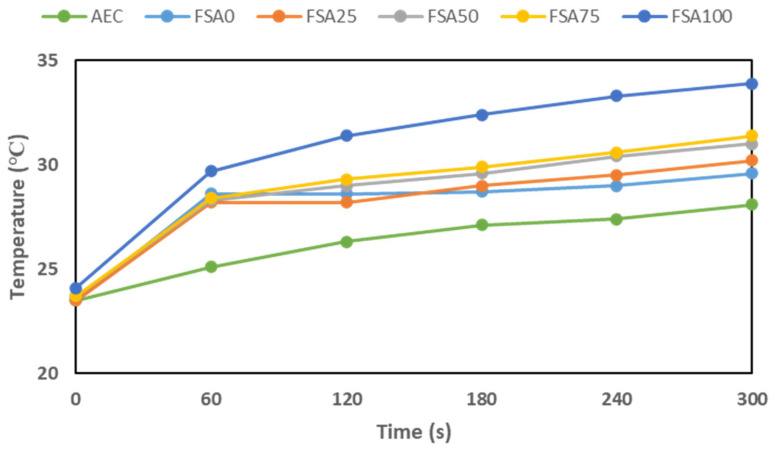
Photothermal performance of the geopolymer after exposure to solar irradiance of 1 kW/m^2^.

A photothermal experiment was performed on geopolymers formulated with varying proportions of fine aggregate and FSA (Table 3). The temperature change was measured after exposure to a solar irradiance of 1 kW/m^2^. The difference in temperature (Δ*T*) is visually presented in Figure 11 and summarized in Table 4.

The AEC mix showed the smallest temperature increase, reaching only 28.1 °C in 300 s. This limited temperature increase can probably be ascribed to the high sand content. Sand, a poor conductor of heat, also has a moderate heat capacity; hence, it resists the transfer of heat from its surface into the bulk and its storage [60]. As heat gets trapped within the material or absorbed very slowly, the temperature will increase rather slowly compared with how it occurs in mixtures containing greater amounts of FSA. This characteristic makes sand-rich mixtures potentially more suitable for applications where thermal insulation is required to reduce heat flow and maintain stable internal temperatures. Intermediate blends with 25–75 wt% FSA showed a moderate increase in temperature, reaching 31.3 °C at 300 s. These mixes depend on the thermal insulating capability of sand rather than the higher thermal conductivity of the FSA components, such as magnetite, to give an intermediate thermal response. Hence, the implication is that by varying the FSA content, the thermal behavior of the geopolymer can be adjusted. These intermediate compositions may be advantageous in applications where moderate heat absorption is beneficial, such as energy-efficient building materials that help maintain stable indoor temperatures without excessive heat gain.

In contrast, the mix composed of 100% FSA had the highest photothermal response, with a surface temperature increase of +9.8 °C after 300 s as compared to its initial temperature. This increase is considerably greater than that of the control AEC mix, indicating that FSA plays a significant role in improving solar energy absorption. Temperature increases of similar magnitudes have been reported based on conductive or darker colored cementitious composites that include magnetite or carbonaceous fillers [61,62]. The temperature of the material appeared to reach approximately 26.2 °C within the first 60 s and then continued heating steadily, suggesting the potential utility of this mix for systems that require thermal mass or a thermal storage capacity, such as passive solar heating.

### 3.5. Changes in the Geopolymer Mass and Strength Under Acid Attack

**Figure 12:**
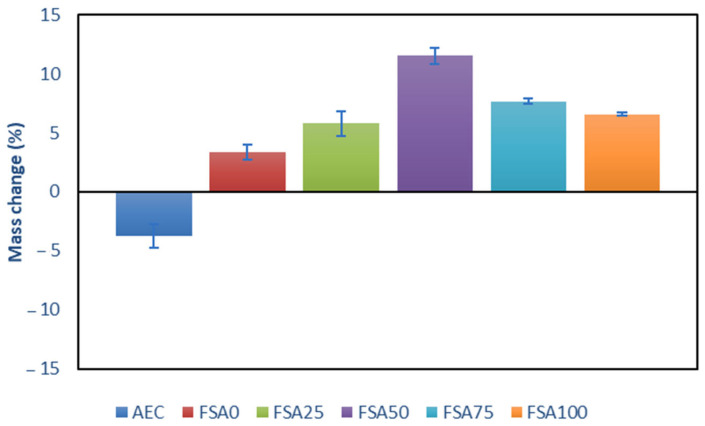
Mass changes of the AEC and geopolymer samples subjected to 28 days of 10% HCl immersion (positive, gain; negative, loss).

The mass change comparison after 28 days of 10% HCl immersion between AEC and geopolymer incorporating varying levels of FSA (0–100%) as a sand replacement is shown in Figure 12. AEC had a 3.75% mass loss, whereas all the geopolymer mixes showed a mass gain, with the highest increase observed for the 50% FSA replacement.

The weight reduction in AEC reflects its vulnerability to acid attack, primarily due to the breakdown of C-S-H into gypsum and silica hydroxide [63]. Acid exposure leads to the leaching of Ca^2+^ ions, which in turn leads to the production of soluble salts and decalcification of the matrix [64], thereby contributing to material deterioration and a loss of material. In contrast, all the geopolymer samples exhibited an increase in weight, with the highest gain (11.51%) at 50% replacement of the aggregate. Wang et al. [65] reported a similar gain in mass phenomena by immersing a fly ash-based geopolymer in a 5% sulfuric acid (H_2_SO_4_) solution for 56 days, which resulted in a weight gain of 0.8%. The FSA inclusion provides more iron sites that can be exposed for a surface reaction with H^+^ and Cl^−^ ions to form insoluble products, such as FeCl_2_, FeCl_3_, and iron oxide films, which could precipitate in pores and surfaces [66]. Although bauxite residue contains iron oxides, the iron oxides primarily exist in stable phases, such as hematite, which are less reactive than the spinel minerals (hercynite, magnetite, and ilmenite) in the FSA. The result is that the FSA0 mix produces none of the protective pore-filling or passivation effects exhibited in acidic environments. Consequently, the FSA0 mix did not exhibit the same protective pore-filling or passivation effects under acidic conditions. The reaction products formed by the reactive phases of the FSA likely account for the observed weight gain and reduced porosity, as insoluble iron compounds and hydroxides of titanium and aluminum precipitate in pores. This secondary phase formation can refine the microstructure and improve resistance to acid penetration.

**Figure 13:**
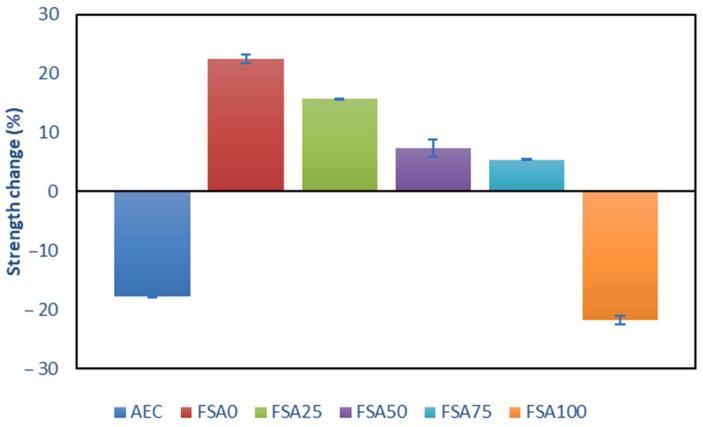
Strength changes of the AEC and geopolymer samples immersed in 10% HCl for 28 days (positive, gain; negative, loss).

To further verify the behavior of the AEC and geopolymer samples subjected to a 10% HCl solution for 28 days, additional strength testing was performed. The average compressive strength results are shown in Figure 13. The AEC and 100% FSA mixes lost strength, whereas partial replacement with 0–75% FSA increased the compressive strength.

The strength reduction in the AEC can be attributed to the acid-induced leaching of calcium phases, which led to structural weakening by increasing porosity [67]. Conversely, geopolymers are more resistant to acid due to their lower calcium content and more chemically stable aluminosilicate gel, which has the tendency to be resistant to leaching in acidic media [68]. The geopolymer samples with lower-to-medium FSA contents (0–75%) showed strength gains after exposure to HCl, with the 0% FSA sample having the highest increase (22.37%). This is perhaps due to continued densification or alkali activation via secondary gel formation upon exposure to a low pH environment. The latter was confirmed by Park et al. [69], who used phosphoric acid (H_3_PO_4_) as the main activator in a metakaolin-based geopolymer. The strength gains decreased with higher FSA contents, likely due to the reduced SiO_2_ available from sand and the increased number of acid-reactive sites, which may compromise the matrix integrity. These intermediate mixes maintain a balance between reactive binder phases and aggregate stability, possibly enabling further alkali activation in acidic media, which improves strength over time [70].

On the other hand, a strength loss of 21.7% was recorded for a 100% FSA replacement, despite the mass gain. This suggests that beyond a certain FSA threshold, acid penetration can compromise the structure instead of reinforcing it. The modified aluminosilicate gel in FSA100 after chloride attack can lead to lower chemical resistance, possibly due to decalcification and the formation of additional mineral products [71], which cannot be overcome through the limited densification of iron-based products. The reduction in strength at 100% FSA loading is not entirely due to a lack of matrix cohesion, and replacing siliceous sand with the Fe-rich spinel aggregate may not only reduce silicon availability for geopolymerization and lower the Si/Al ratio but also result in the limited formation of a continuous aluminosilicate gel network.

### 3.6. XRD Analysis

**Figure 14:**
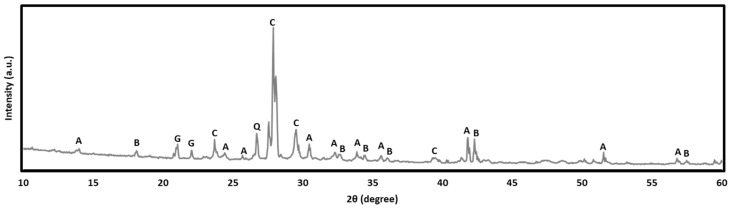
XRD patterns of air-entrained Portland cement-based concrete (A—alite, B—belite, C—calcite, G—gypsum, and Q—quartz).

The XRD patterns of AEC after 28 days of oven curing (Figure 14) revealed the predominant presence of alite (C3S), belite (C2S), calcite (CaCO_3_), and gypsum (CaSO_4_.2H_2_O), alongside with minor trace amounts of quartz (SiO_2_), a trend consistent with previous reports [72]. The presence of gypsum could increase the porosity of concrete and makes it susceptible to microcrack propagation [73].

**Figure 15:**
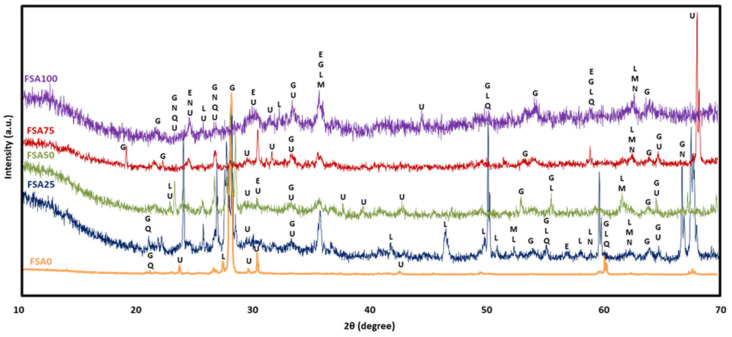
XRD patterns of the geopolymer with different percentages of FSA as a sand replacement (E—esseneite, G—gismondine, L—laumontite, M—magnetite, N—NaFeO_3_, Q—quartz).

The diffractogram of the geopolymers is shown in Figure 15. It is evident that Portland cement and geopolymers exhibit distinctive reaction products. The patterns of the mixtures indicate the possible presence of zeolitic phases, which may form during the alkali activation process.

The XRD analysis revealed that the geopolymers with different FSA replacement percentages were predominantly amorphous in nature, which is attributed to the low intensity of the ordered mineral structure. On the contrary, FSA0 exhibited clearly defined aluminosilicate gels, such as gismondine (CaAl_2_Si_2_O_8_.4H_2_O), laumontite (CaAl_2_Si_4_O_12_.4H_2_O), and ussingite (Na_2_AlSi_3_O_8_OH), along with the presence of quartz from the raw material and sand aggregate. The presence of aluminosilicate likely contributes to enhanced acid resistance and structural stability [74]. These phases can buffer acid diffusion through ion exchange mechanisms and physical densification, reducing permeability and pore connectivity [75]. The formation of gismondine, laumontite, and ussingite indicates the partial zeolitization of the geopolymer matrix. These occurrences typically form when excess aluminosilicate species reorganize themselves into a more thermodynamically stable three-dimensional network structure under alkaline conditions [76]. Importantly, the occurrence of these secondary crystalline phases aligns with the observed strength development in FSA-rich mixes, suggesting that they not only reflect the chemical evolution of the system but could also stabilize the matrix microstructure and enhance its mechanical performance over time.

The increase in the FSA content induced a parallel increase in the overall crystalline phases due to the noticeable appearance of the magnetite (Fe_3_O_4_), NaFeO_3_, and esseneite (CaFeAlSiO_6_) phases. The latter indicates partial crystallization of the Al-Si network gels into ferrosialate (-Fe-O-Si-O-Al-O-) phases [77], which implies effective geopolymerization and better chemical stability. This is likely to contribute to both mass and strength retention or a gain in geopolymers with an FSA content of up to 75% sand replacement. Fe-rich aluminosilicate phases have also been reported to have superior durability in sulfate exposure [78]. The unusual NaFeO_3_ formation that is a sodium–iron phases have been reported in the literature, for instance when lateritic aluminosilicate was activated with phosphoric acid and subsequently calcined [79].

On the other hand, the FSA100 sample showed a decline in mechanical performance (approximately 21.7%), despite gaining mass. This can be attributed to the lack of a silica phase, which is crucial for durable alumino-silicate gel formation. Excess FSA might disrupt the formation of a continuous matrix network while also creating more chemically reactive Fe-based sites that could be less stable under acid attack. Moreover, high crystallinity could reduce the flexibility of the matrix to accommodate chemical interactions [80], thereby lowering mechanical performance. In contrast, the AEC showed a 3.75% mass loss and 17.75% strength reduction after immersion in 10% HCl, likely due to the leaching of calcium-rich hydration products (e.g., portlandite and C-S-H) that are vulnerable in acidic environments [81]. The absence of a stable Al-Si network and the dominance of acid-sensitive portlandite and C-S-H gels in OPC-based concrete result in greater susceptibility to chemical degradation. The acid resistance of geopolymers is superior to cement owing to their high alkali content [82]. Meanwhile, the acid resistance of conventional concrete relies heavily on its protective layer [83].

### 3.7. Microstructural Changes in the Geopolymer Samples Under Acid Attack Through SEM Analysis

**Figure 16:**
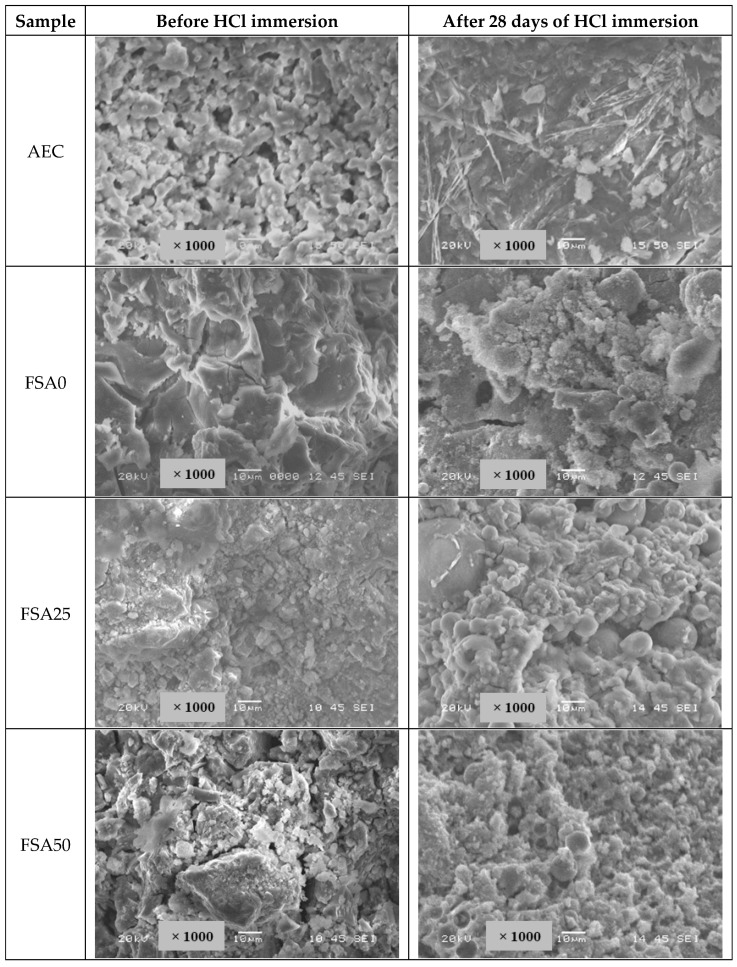

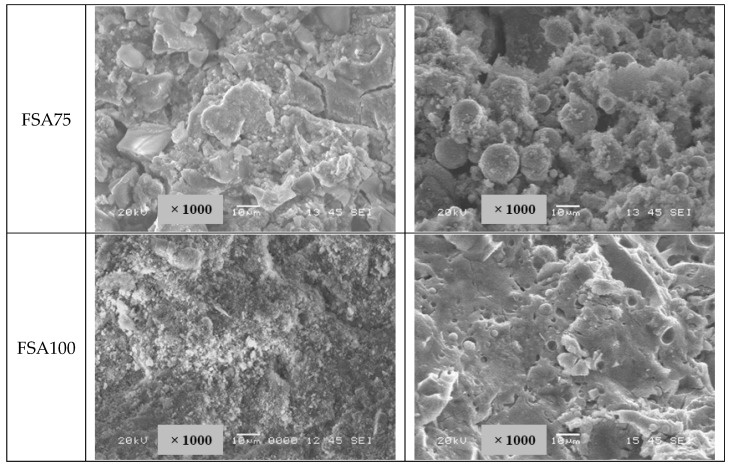
Microstructural changes in the AEC and geopolymer samples before and after exposure to 10% HCl for 28 days.

The SEM images of the conventional concrete and alkali-activated matrix taken before and after acid immersion are shown in Figure 16. The images taken before exposure displayed a dense network of hydration products in the AEC and the aluminosilicate gel framework of the geopolymer. The microstructure showed clear changes after acid exposure, indicating material degradation. The needle-like ettringite crystals that formed during cement hydration were still observed in the AEC mix even after acid immersion, indicating incomplete decomposition. Their persistence may contribute to structural deterioration and mass loss under acidic conditions. This phase is unstable and decomposes when the pH value decreases more than 11.5–12, which was the case upon acid exposure [84], leading to material degradation.

Meanwhile, the SEM images of the geopolymer present considerable differences in morphologies with different FSA contents. Aluminosilicate-based matrix exhibits more acid resistance than normal OPC hydration products [85]. The geopolymer samples with FSA contents of up to 75% exhibited the formation of bubble-like particles. Soaking the Fe-rich material prepared from bauxite residue and the heavyweight aggregate caused the leaching of Fe^2+^ [86]. This was followed by a reaction with Cl- ions to develop FeCl_3_ [87], which could result in a denser framework by sealing the microcracks in the geopolymers prior to acid exposure. Moreover, the incorporation of iron-rich aggregates promotes a finer pore structure, as the FSA has reduced the connectivity of large pores in the mix, which limits the area of concentrated stress under a load. Thus, the stronger mechanical properties observed can be ascribed to a combination of these microstructural and chemical factors, rather than only the densification effect. However, FSA100 exhibited hairline cracks, which also likely to trigger crack propagation and increased porosity at augmented FSA contents, which could makes the material more prone to acid penetration and deterioration [88]. This highlights the importance of optimizing the amount of the heavyweight aggregate to balance the functional benefits with structural durability.

## 4. Conclusions

This study investigated the durability, microstructural evolution, and multifunctional performance of a GGBFS–bauxite residue-based geopolymer incorporating FSA as a partial-to-full replacement for silica sand. The results demonstrate that an intermediate amount of heavyweight filler replacement achieved the most favorable balance between early and long-term compressive strength and microstructural integrity. At these levels, the coexistence of reactive aluminosilicate phases and heavyweight aggregates contributed to densification and strength development.

The FSA-enhanced geopolymer exhibited promising electrical performance by significantly reducing resistivity with an augmented FSA content, reaching as low as 42 Ω·m at 100% sand replacement, supporting the prospect of using FSA-enhanced geopolymers in electrically conductive infrastructure. The photothermal performance assessment further highlighted the role of Fe-rich phases in enhancing the thermal response of geopolymers. Higher FSA contents enhanced solar heat absorption and retention under simulated sunlight, enabling tailored thermal behavior.

Acid exposure testing revealed that in addition to functional properties, the geopolymers exhibited outstanding acid resistance at an FSA content up 75%, where the mixtures in fact gained mass rather than losing it, differing from the conventional AEC, which experienced material degradation. The weight increase in the alkali-activated system was most likely due to the acid reaction, FSA surface reactions forming passive iron compounds, and ionic bonding, which help in densification. These beneficial effects were observed only within an optimal range of heavyweight fillers. The XRD analysis confirmed the formation of aluminosilicate crystalline phases, such as gismondine, laumontite, and ussingite, in the geopolymers, which are presumably responsible for strength retention and chemical resistance to degradation. However, while 100% FSA replacement provided optimal electrical and thermal performance, it also showed reduced acid resistance. This highlights the need to balance the FSA content to achieve multifunctionality without compromising long-term structural stability.

The use of the bauxite residue and GGBFS not only provides functional and durability advantages but also provides significant sustainability benefits. By referring to sustainable waste material, this approach reduces disposal issues and simultaneously lowers the reliance on Portland cement. This research focused on the engineering properties of the waste-derived materials, but a full life cycle assessment is beyond the scope of this study. However, the combined benefits of waste valorization and avoiding emissions related to clinker production indicated that FSA-enhanced geopolymers may be considered among the alternative solutions for sustainable construction. Overall, this study validates the potential of these systems as multifunctional construction materials. These composites provide a sustainable pathway for advanced infrastructure applications, such as snow-melting pavements and self-heating concrete. Future research should therefore include a direct measurement of Young’s modulus to provide a more comprehensive understanding of the mechanical performance of FSA-enhanced geopolymers, examine their long-term performance under real environmental conditions, and investigate scalability for field applications to further unlock their infrastructure readiness and environmental benefits.

## Figures and Tables

**Table 1:** Chemical compositions of the bauxite residue and GGBFS.

Oxide (%)	Bauxite Residue	GGBFS
SiO_2_	11.1	33.8
Al_2_O_3_	17.2	9.9
Fe_2_O_3_	44.4	1.1
MgO	0.05	5.9
CaO	2.61	46.2
Na_2_O	6.44	0.6
K_2_O	0.06	-
LOI	11.9	-

**Table 2:** Mix proportions of the air-entrained concrete that conformed to CSA A23.1 [22].

Sample	Portland Cement (kg/m^3^)	Fine Aggregate (kg/m^3^)	Coarse Aggregate (kg/m^3^)	Water (kg/m^3^)	Sika Air 60 (kg/m^3^)	Sika Viscocrete (kg/m^3^)
AEC	420	950	804.3	157	0.15	3.12

**Table 3:** Mixtures of the geopolymer samples with different aggregates.

Sample	Bauxite Residue (kg/m^3^)	GGBFS (kg/m^3^)	Fine Aggregate (kg/m^3^)	Fine Magnetite (kg/m^3^)	NaOH (kg/m^3^)	Na_2_SiO_3_ (kg/m^3^)	Graphite (kg/m^3^)
FSA0	384	576	1920	0	112	224	9.6
FSA25	384	576	1440	480	112	224	9.6
FSA50	384	576	960	960	112	224	9.6
FSA75	384	576	480	1440	112	224	9.6
FSA100	384	576	0	1920	112	224	9.6

**Table 4:** Changes in the geopolymer temperature at different time intervals under photothermal exposure.

Sample	*T*_min_ (°C)	*T*_max_ (°C)	Δ*T* (°C)
AEC	23.5	28.1	4.6
FSA0	23.5	29.6	6.1
FSA25	23.5	30.2	6.7
FSA50	23.7	31	7.3
FSA75	23.7	31.3	7.6
FSA100	24.1	33.9	9.8

## Data Availability

The original contributions presented in this study are included in the article. Further inquiries can be directed to the corresponding authors.

## Abbreviations

The following abbreviations are used in this manuscript:

FSA	Fe-rich Spinel Aggregate
GGBFS	Ground Granulated Blast Furnace Slag
AEC	Air-entrained Concrete
OPC	Ordinary Portland Cement
XRD	X-ray Diffraction
SEM	Scanning Electron Microscopy
XRF	X-ray Fluorescence
CSA	Canadian Standards Association
CSH	Calcium–Silicate–Hydrate
HCl	Hydrochloric Acid
